# Correction of Condylar Displacement of the Mandible Using Early Screw Removal following Patient-Customized Orthognathic Surgery

**DOI:** 10.3390/jcm10081597

**Published:** 2021-04-09

**Authors:** Won-Seok Jang, Soo-Hwan Byun, Seoung-Won Cho, In-Young Park, Sang-Min Yi, Jong-Cheol Kim, Byoung-Eun Yang

**Affiliations:** 1 Division of Oral & Maxillofacial Surgery, Hallym University Sacred Heart Hospital, Anyang 14066, Korea; wionnk@naver.com (W.-S.J.); purheit@daum.net (S.-H.B.); kotneicho@gmail.com (S.-W.C.); queen21c@gmail.com (S.-M.Y.); ddskjc@hanmail.net (J.-C.K.); 2 Graduate School of Clinical Dentistry, Hallym University, Chuncheon 24252, Korea; park.iy2875@gmail.com; 3 Institute of Clinical Dentistry, Hallym University, Chuncheon 24252, Korea; 4 Division of Orthodontics, Hallym University Sacred Heart Hospital, Anyang 14066, Korea; 5 Department of Implant Dentistry, Daegu Mir Dental Hospital, Daegu 41940, Korea

**Keywords:** post-surgical malocclusion, patient-customized orthognathic surgery, condylar sag, screw removal, temporomandibular joint

## Abstract

Objective: Orthognathic surgery (OGS) is a surgical intervention that corrects dentofacial deformities through the movement of maxillary and mandibular segments to achieve adequate masticatory function, joint health, and facial harmony. However, some patients present with occlusal discrepancies, condylar sag, and/or temporomandibular disorders after OGS. Various methods have been employed to solve these problems after surgery. This study aimed to evaluate the effectiveness of early screw removal in patients with occlusal discrepancies after OGS using three-dimensional cone-beam computed tomography (CBCT). Methods: In 44 patients with dentofacial deformities, patient-customized OGSs with customized plates were performed to correct facial deformities using customized guides with computer-aided surgical simulation. Of the 44 patients, eight patients complained of occlusal discrepancies and temporomandibular disorders after OGS. These eight patients underwent screw removal under local anesthesia around four weeks. The temporomandibular joint spaces at three time points (pre-surgical, post-surgical, and after screw removal) in the sagittal and coronal planes were compared using CBCT. Results: Eight patients showed an increase in joint space on CBCT images immediately after surgery (T1), but after early screw removal (T2), these spaces almost returned to their pre-surgical state, and the temporomandibular joint problem disappeared. Conclusions: The removal of screws located in the distal segment under local anesthesia between three and four weeks post-surgically may be a treatment option for patients with post-OGS occlusal discrepancies, condylar sag, and/or temporomandibular disorder.

## 1. Introduction

Orthognathic surgery (OGS), also known as corrective jaw surgery, is designed to correct conditions of the jaw and face related to structure, growth, airway issues, including sleep apnea, impaired masticatory function, joint disorders, skeletal malocclusion, and/or other orthodontic problems that cannot be easily treated with conventional orthodontic bracket treatment, as well as the broad range of facial imbalances, disharmonies, and asymmetries where correction can be considered to improve facial esthetics. 

The two commonly used mandibular orthognathic surgery includes intraoral vertical ramus osteotomy (IVRO) and sagittal split ramus osteotomy (SSRO). Both IVRO and SSRO have the potential for postoperative condylar displacement [1]. Rotational displacement could cause condylar displacement and temporomandibular joint (TMJ) disorder. IVRO is preferred because it causes less rotational displacement than SSRO [2]. Furthermore, IVRO has been used to treat patients with TMJ disorder [3]. However, because the contact area between the proximal and distal segment is small, an intermaxillary fixation (IMF) period of 6 weeks is recommended [4]. The need for IMF in IVRO also raises concerns regarding immediate postoperative airway management, and patients on IMF require a liquid diet during the fixation period. The jaw immobility in IVRO further affects patient quality of life. For this reason, SSRO is more prevalent in the majority of orthognathic centers in many parts of the world [5]. SSRO leads to wider bone contact between the proximal and distal segments; hence, it contributes to favorable healing and a short maxillomandibular fixation period [6,7]. Additionally, SSRO can be used to treat both mandibular prognathism and retrognathism because it can be used both for the forward and backward movements of the segments. However, postoperative complications, such as nerve injury, relapse, TMJ dislocation, condylar sag [8], and condylar resorption, have been reported [9,10,11]. As it requires a relatively short period of intermaxillary fixation, rigid fixation is commonly used. However, rigid fixation may cause displacement of the condyle from the fossa, which can result in relapse [12]. After SSRO, positional changes in the TMJ can occur as a side effect, leading to occlusal discrepancies. Relapse and occlusal discrepancies occur following condylar sag if the proper condyle–fossa relationship is not maintained during surgery or if the correct placement of the rigid fixation is not achieved. Thus, many studies have suggested ways to prevent condylar sag or establish the correct position of the condyle intraoperatively [13,14,15]. However, despite various methods to prevent condyle sag during OGS, there may be cases in which problems with the condyle–fossa relationship occur after OGS. There is no established definitive treatment for condyle displacement after OGS. Few studies have suggested early plate removal. Recently, Yoko et al. reported condylar positional changes in the TMJ after early plate removal, but the study used the TMJ Schuller method to evaluate the TMJ space [16]. This method can be used to evaluate the TMJ space, but it has limitations in that it cannot evaluate the degree of rotation of the condyle and the volume of the TMJ space. In addition, the operation time is increased, and a wide mucosal incision is required to remove the entire plate. Hence, this study used a technique of early screw removal in the distal segment around four weeks after OGS and three-dimensional cone-beam computed tomography (CBCT) to compare the positional changes in the mandibular condyle for the accurate evaluation of the joint space of the TMJ. 

We recently reported that patient-customized OGS using a customized titanium plate could be performed with an accuracy similar to that obtained by virtual surgery [17]. However, according to a recent study, virtual surgery is sensitive (100%), but the specificity is reported to be low (51.6%) in reflecting the proximal and distal segment interference of the mandibular bone in actual surgery [18]. In our recent study, the condyle position was adjusted to establish the optimal relationship between the proximal and distal segments during mandibular virtual surgery. However, there were cases wherein the condyle–fossa relationship after the operation was not similar to that after the virtual surgery [19].

Hence, the purpose of this study was to evaluate the positional changes in the TMJ and the effectiveness of early screw removal using CBCT after patient-customized OGS in patients with occlusal discrepancies.

## 2. Materials and Methods

Forty-four patients with dentofacial deformities visited the university hospital and were treated by SSRO with or without Lefort I osteotomy from February 2015 to February 2019 using a customized titanium plate. The SSRO was performed according to Obwegeser’s method, using customized guides, plates, and proximal segment positioning devices (PSPD) [19] during the surgery. Before the surgery, patients’ photographs, CBCT scans, facial scans, model scans, and information on the occlusal relationship data were obtained. All data were imported and merged with the CBCT data, and the surgical procedures were simulated virtually with program (FaceGide^®^ (MegaGen Co., Daegu, Korea)) (Figure 1). 

The preparation and procedure for surgery were as follows. A clinical examination and three-dimensional (3D) data acquisition (CBCT image and an image of the patient’s dentition using a 3D model scanner) were performed, and a composite 3D virtual model (Virtual Face) was built. We quantified the deformity via 3D anthropometric analysis, and surgical simulation, including osteotomy and repositioning of the bone segments, was then performed. The osteotomy guides, customized fixation plates, surgical wafers, and PSPD were designed and manufactured. A virtual surgical plan and the associated materials were delivered to the operating room. 

During the actual surgery, the authors used a customized 3D-printed osteotomy guide and a customized milled plate [17]. The final position of the proximal segment was determined through virtual surgery, and the distance between the condyle and mandibular fossa and the degree of yaw control for both condyles was measured in the same manner as that used to perform measurements on the CBCT images. In the operating room, after performing the SSRO using an osteotomy guide, the position of the proximal segment that was set in the virtual surgery was reproduced using the PSPD, and both segments were fixed with a customized miniplate [19]. IMF was performed for one or two weeks using light elastics. After the surgery, eight patients had occlusal discrepancies or condylar sag and complained of discomfort around the TMJ. Bilateral or unilateral removal of the screw in the customized plate of the distal segment of the mandible was performed in these patients approximately four weeks after patient-customized OGS under local anesthesia (Figure 2).

CBCT scans were obtained at several time points: pre-surgical (T0), post-surgical two weeks (T1), and 4 to 5 months after screw removal (T2). CBCT scans were obtained using Asahi Alphard 3030^®^ (Asahi Roentgen Ind., Co. Ltd., Kyoto, Japan) in C-mode with an imaging volume of 200 × 178 mm and a voxel size of 0.39 mm. The scanning parameters were fixed at 80 kVp, 5 mA, and 17 s for all patients. The evaluation of TMJ positional changes was performed before and after the surgery using CBCT, OnDemand 3D software (Cybermed, Seoul, Korea), and Checkpoint software (Stratovan, Davis, CA, USA) (Figure 3). Digital imaging and communications in medicine (DICOM) images were reconstructed using OnDemand3D software, which were oriented along the Frankfort horizontal plane in reference to the right porion, nasion, and left orbitale.

We chose a plane of reference at the left external carotid canal in the axial view, which was a line from the innermost point to the outermost point of the condyle parallel to the sagittal plane. After that, we set a plane perpendicular to the line at its midpoint. The superior joint space (SJS) was defined as the distance measured from the top of the condyle to the deepest point of the glenoid fossa in the plane. Tangent lines were drawn to the most prominent points on the condyle’s anterior and posterior aspects from the deepest point of the glenoid fossa. The anterior joint space (AJS) was defined as the perpendicular distance between the most prominent point on the anterior aspect and the glenoid fossa. The posterior joint space (PJS) was defined as the perpendicular distance between the most prominent point on the posterior aspect and the glenoid fossa. The angle of the condyle or the condylar angle was defined as the angle between the line from the innermost point to the outermost point of the condyle parallel to the sagittal plane and the sagittal plane (Figure 4).

Joint space volume was calculated using the Checkpoint software. The 3D skull images were reconstructed in the software using DICOM data. The postglenoid process, articular eminence, and entoglenoid process were set as reference points, and the plane passing through the three points was set as the reference plane. The volume of the space between the glenoid fossa and the condyle above the reference plane was defined as the joint space volume (Figure 5).

Statistical analysis was performed using the Friedman test, and the Bonferroni correction posthoc test was conducted for statistical analysis. The data were subsequently analyzed using Statistical Package for Social Sciences (version 26.0, IBM Corporation, Armonk, NY, USA), in which *p*-values less than 0.05 were considered to indicate significance. The study protocol was approved by the Hallym University Sacred Heart Hospital Institutional Review Board (IRB No. 2020-07-009-001). The IRB approved this retrospective study, and all patient data were anonymized and de-identified before the analysis.

## 3. Results

Of the eight patients, seven patients underwent SSRO + Le fort I osteotomy, and one patient underwent only SSRO. The average period from patient-customized OGS to screw removal was 25.3 ± 3.2 days (Table 1). 

The average anterior joint space distances at T0 and T1 were 1.58 mm and 3.16 mm, respectively, which showed a statistically significant difference (*p* = 0.002). After removal of the screw (T2), the distance decreased to 1.79 mm, which showed a significant statistical difference (*p* = 0.004) when compared with the distance after the surgery (T1). There was no statistically significant difference between T2 and the pre-surgical distance (T0) (*p* = 1.000). The average volume of superior and posterior joint spaces showed significant statistical differences among T0, T1, and T2. The average condylar angle measurement showed a significant statistical difference only between T0 and T1. After removing the screw, the condylar angle at T2 was greater than that at T0, but the difference was not statistically significant (*p* = 0.266). Two patients showed significant changes in the condylar position on one side only. Therefore, the screws of the joints without problems were not removed, and a total of 14 joints were investigated and processed for statistics (Table 2).

## 4. Discussion

OGS corrects the irregularities in the jaw bones and realigns the jaws and teeth to improve their function and the patient’s facial appearance. OGS is associated with several complications, a significant one being mandibular condyle misplacement. Our findings suggest that early screw removal could be an alternative treatment option in patients with occlusal discrepancies and condylar sag after SSRO. Nowadays, OGS is commonly performed for orthodontic and cosmetic purposes and is gaining popularity. Several types of orthognathic surgeries are performed in the mandible, and SSRO is a common method used to correct arch relationships. SSRO has several advantages over IVRO, including wider bone contact between the proximal and distal segments [20,21]. However, occlusal discrepancies might be encountered after SSRO. Correction of moderate and severe occlusal discrepancies is challenging with elastic traction and post-surgical orthodontic treatment. Moreover, if rigid fixation is used during SSRO, improper positions of the mandibular segment can cause TMJ dysfunction and discomfort.

In this study, after removing the screws, the joint space was almost restored to pre-surgical condition, but the condylar angle was significantly greater than the original value. This change in angle could be attributed to the condyle yaw control [19] in virtual surgery and its reflection in the design and fabrication of customized plates used in the actual surgery. Therefore, even after the screws were removed, the condylar angle was greater than the pre-surgical value. In this study, only the screws of the distal segments were removed, retaining the screws of the proximal segment and the plate, in patients with occlusal discrepancies. Although rigid fixation by plates was lost, stable bone healing was obtained through intermaxillary fixation using the elastics. The formation of bone calluses is observed in the normal fracture healing process, between 2 and 3 weeks [22,23]. Nine days after fracture, the chondrocytes of the soft callus adjacent to the woven bone of the hard callus begin to elongate [24] and adhere to the customized titanium plate from all sides. We attempted to preserve this callus. The customized plate was also preserved on the bone surface to prevent the proximal segment’s movement toward the bucco-lingual side. Some movement between the distal and proximal segments was possible by removing only two screws from the distal segment. Removal of only the screws of the distal segment has several advantages over the removal of the entire plate. The incision for screw removal is small, requires less time, is less invasive, and offers limited interference with the initial bone healing process. Additionally, during the follow-up period, the screws of the plate in the proximal segment showed no sign of inflammation, and no side effects such as fracture and/or malunion were observed.

On average, the joint space volume had increased by 30% at T1 compared to that at T0, which had decreased after screw removal. Based on these results, we believe that early screw removal can be used for condylar repositioning and improving occlusal relationship when the postoperative joint space volume has increased by more than 30% compared with the pre-surgical values. We attempted to predict the postoperative intermaxillary relationship through a virtual simulation before surgery and then performed patient-customized OGS based on the virtual stimulation, but some patients showed midline deviation after the surgery. These patients showed changes in the condylar space; the midline deviation was corrected, and the joint space returned to the pre-surgical state after screw removal. However, after screw removal, CBCT was performed after five months (T2), and since orthodontic forces were applied during this period, the possibility that the orthodontic forces had a role in midline correction cannot be excluded. 

Patient-customized OGS using customized fixation plate after the virtual simulation is a more effective strategy than using CAD-CAM splints alone [25]; however, some errors in the diagnosis and treatment planning may occur due to limitations in manipulating 3D data on a 2D screen [26]. In this study, we used customized guides, plates and PSPDs for OGS. Patient-customized OGS has an acceptable accuracy, similar to that of virtual surgery [17]. However, side effects such as occlusal discrepancies after customized guided surgery are still evident. Errors in accuracy can occur in all treatment steps, such as model scanning, insertion and integration of DICOM and stereolithography files, determination of coordinates in the 3D environment, and fabrication of osteotomy guides customized plates. Minor errors in each step accumulate and could lead to accuracy errors [17]. Kwon also reported that computer-assisted 3D virtual model surgery and 3D-printed intermediate occlusal splint were useful but not completely accurate; random errors are inevitable [27]. This is because the humans’ masticatory movement is a mutually complex interaction between the nervous and musculoskeletal systems [28] while the osteotomy guide is based on static occlusal and skeletal relationships only.

In this study, eight out of forty-four patients who underwent patient-customized OGS were diagnosed as having problems with the condyle–fossa relationship. Epker et al. reported that the three reasons to maintain the pre-surgical condylar position in OGS are to ensure the stability of surgical results, reduce the adverse effects on the TMJ, and improve masticatory function [29]. However, Ueki et al. suggested that the pre-surgical position of the condyle was not the desired post-surgical position in OGS [30]. Based on Ueki’s argument, the post-surgical condylar position that could not be adjusted in conventional OGS was adjusted to a new position planned for some patients [19] at patient-customized OGS using virtual surgical planning and various customized materials. However, since the post-surgical condylar position was not always the same as the position set in the virtual surgery, eight patients in this study were found to have problems with the condyle–fossa relationship. We did not judge these cases as severe displacement, but screw removal of the distal segment was performed to facilitate post-surgical orthodontic treatment. Therefore, further studies will be needed to determine the appropriate condylar position after surgery.

Changes in disc position were not evaluated in this study. However, OGS does not affect the disc position [31,32], although it is affected by the position of the condyle [33]. Louis et al. noted that OGS could change the condylar position in the fossa, which can change the magnitude and direction of the forces in the TMJ. In most patients, the condyle and fossa change and adapt to the changing forces acting in the TMJ. However, condylar resorption can occur when the altered forces exceed the limit of the TMJ. Since our data were based on the anatomical points in the condyle and fossa only, this condylar resorption may have been included in the data [34]. 

Hadjidakis et al. reported that the bone remodeling cycle consists of three consecutive phases: resorption, reversal, and formation. The stages of the remodeling cycle have different lengths. Resorption probably continues for about two weeks, and the reversal phase may last up to 4 or 5 weeks, while formation can continue for four months until the complete formation of the structural unit of new bone [35]. Therefore, we performed the removal of the screws of the distal segment between 3 and 5 weeks after SSRO. Since hard callus remodeling begins three weeks after fracture [36], we estimated that the same effect as the result of this study would not been obtained if the screw or plate was removed about four weeks after surgery.

The limitation of this preliminary study is that the number of patients was relatively small. This is because patient-customized OGS using virtual surgery and customized plates was performed in most patients exactly as planned. Therefore, few patients exhibited abnormal condyle–fossa relationships. Furthermore, only a limited, albeit homogenous, patient collective was included; for example, the condyle–fossa relationship was relatively similar to that before OGS, but patients with a compromised bite were also included. Therefore, this study’s results cannot be generalized to all patients, and follow-up studies will be needed with a larger number of patients. Another limitation of the study was the lack of a control group. This study was conducted on cases where the volume between the condyle–fossa was not more than 30% compared to the pre-surgical state; a follow-up study is needed for cases with a larger displacement in the conventional OGS.

However, to our knowledge, this is the first study to determine the efficacy of resolving TMJ problems after patient-customized OGS by only screw removal while leaving the customized plate. In addition, it provides a methodology to measure the 3D volume of the space between the condyle and fossa. Although the accuracy of OGS using a customized plate has been reported [25,26], surgeons and orthodontic doctors should be aware of the possible errors in surgery due to improper process of collecting patient-related data, processing digital data, and fabrication of patient-customized materials.

## 5. Conclusions

The joint sizes in three areas, condylar angle, and condyle space volume almost returned to pre-surgical values after early screw removal. As a result, these patients showed good outcomes. Early removal of screws under local anesthesia could be a treatment option for patients with postoperative occlusal discrepancies after SSRO. The optimal timing for screw removal is around four weeks postoperatively. The clinician should also be aware that errors in bone positioning may occur despite the patient-customized OGS using virtual surgical planning and various customized materials. Further studies are required to validate these findings while considering bone healing effects between the proximal and distal segments after fixation screw removal.

## Figures and Tables

**Figure 1:**
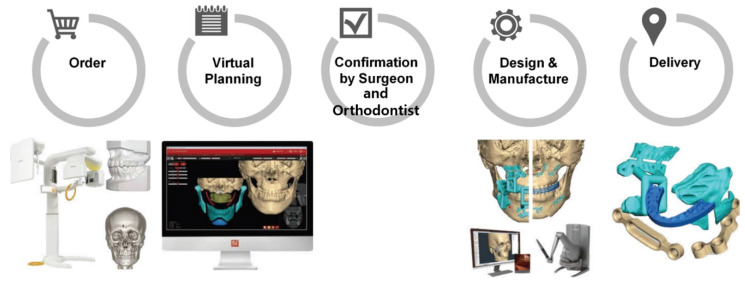
Work process of patient-customized orthognathic surgery with computer-aided surgical simulation system using FaceGide^®^ [17].

**Figure 2:**
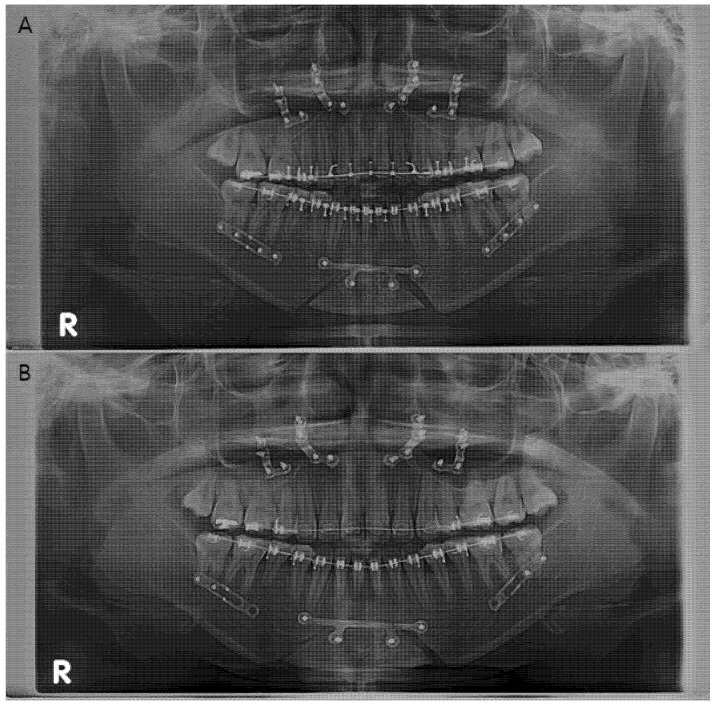
(**A**) Panoramic radiograph of a patient (Patient no. 3) who had undergone orthognathic surgery. (**B**) Panoramic radiograph after early screw removal in the distal segment. “R” on the panoramic radiograph means the right side of the patient.

**Figure 3:**
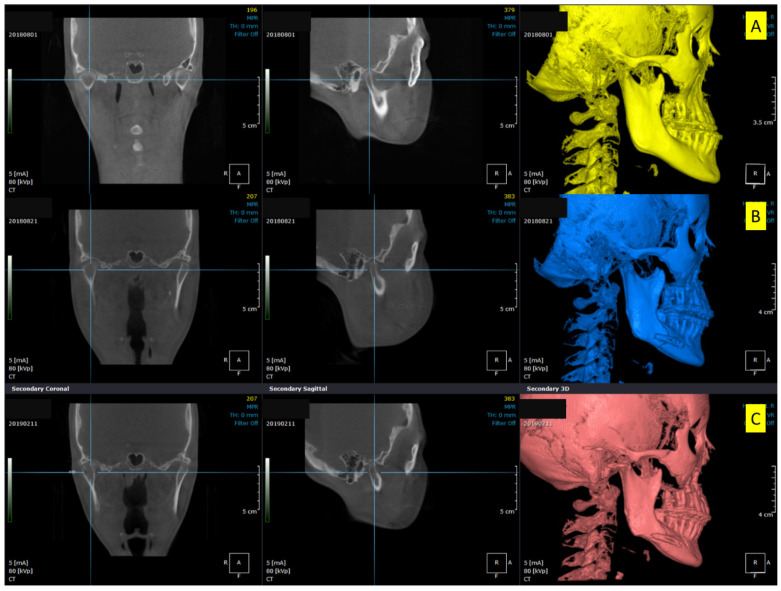
Three-dimensional cone-beam computed tomography scans showing the changes in condylar positions in Patient no. 2. (**A**) Pre-surgical, (**B**) Post-surgical, (**C**) Five months after screw removal. Increased temporomandibular joint space is seen in (**B**). After screw removal, the joint space decreased and was restored to the pre-surgical state.

**Figure 4:**
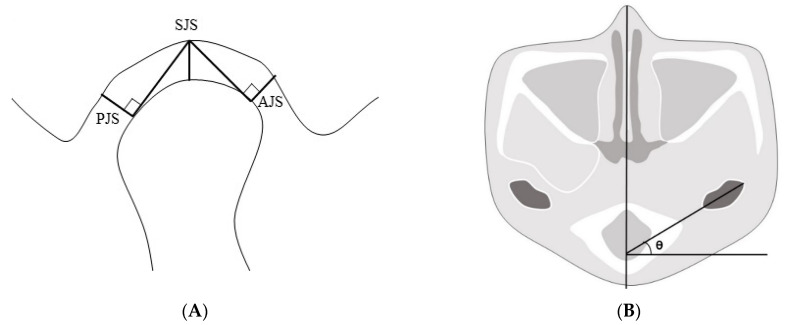
(**A**) The plane of reference in the axial plane at the left external carotid canal with lines from the innermost and outermost points on the condyle. Reference plane perpendicular to the line at the midpoint. Anterior, superior, and posterior joint spaces in the plane (AJS, SJS, and PJS, respectively). (**B**) The angle of the condyle was defined as the angle between the line from the innermost point to the outermost point of the condyle parallel to the sagittal plane and the sagittal plane. AJS: anterior joint space; SJS: superior joint space; PJS: posterior joint space.

**Figure 5:**
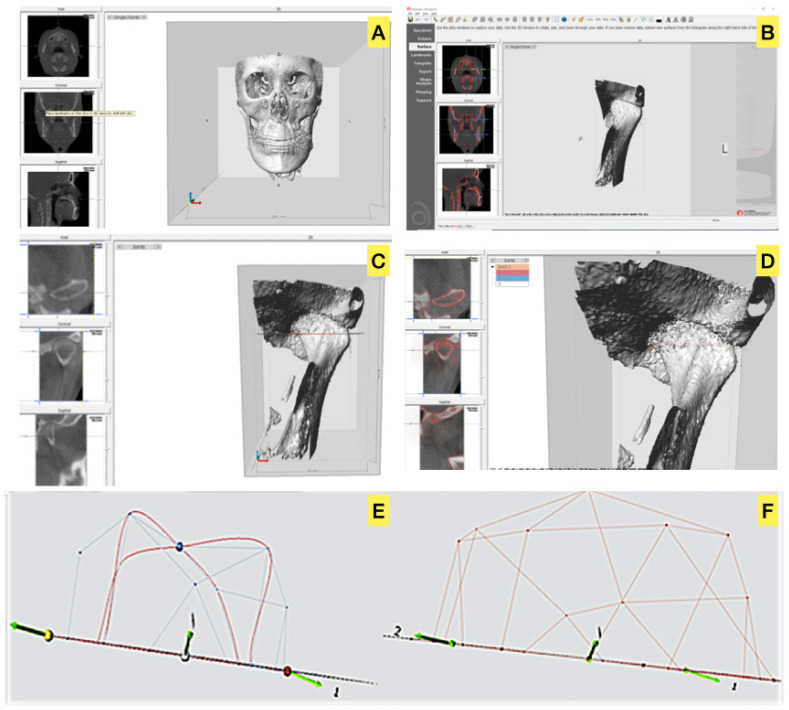
A view on Checkpoint (Stratovan): (**A**). Imported cone-beam computed tomography data exported in digital imaging and communications in medicine format, (**B**). Cropped image of the volume of the left condyle–fossa unit, (**C**). Individual points on the medial and lateral poles of the condylar head, (**D**). Distance measurement between the medial and lateral poles of the condylar head (diameter), (**E**). Volume space of the condylar head above the reference plane, (**F**). Volume space between the reference plane and glenoid fossa. We defined the difference between (**E**) and (**F**) as the joint space volume.

**Table 1:** Patient characteristics and descriptions of the performed surgeries.

Pt. No	Age	Sex	Diagnosis	Surgery	The Period from OGS to Screw Removal (Days)	Removal Site
Pt. 1	26	M	Class III	LFI + SSRO	24	Both
Pt. 2	26	F	Class III	LFI + SSRO	30	Both
Pt. 3	20	F	Class III	LFI + SSRO	30	Both
Pt. 4	55	F	Class III, FA	SSRO	22	Right
Pt. 5	22	M	Class III	LFI + SSRO	25	Left
Pt. 6	18	F	Class III, FA	LFI + SSRO	24	Both
Pt. 7	20	F	Class III, FA	LFI + SSRO	25	Both
Pt. 8	20	M	FA	LFI + SSRO	22	Both

Class III, Skeletal Class III malocclusion; FA, Facial Asymmetry; LFI, Lefort I osteotomy; SSRO, Sagittal Split Ramus Osteotomy; OGS, orthognathic surgery; Pt, Patient; M, Male; F, Female.

**Table 2:** Change in the average size of the AJS, PJS, SJS, angle, and volume at T0, T1, and T2 with the statistical analysis.

	T0	T1	T2	H	*p* ^(1)^	df	B ^(2)^
	Mean ± SD	Mean ± SD	Mean ± SD				
AJS (mm)	1.58 ± 0.25	3.16 ± 1.58	1.79 ± 0.79	15.11	0	2	T1 > T0,T2
PJS (mm)	1.93 ± 0.64	3.93 ± 1.96	2.36 ± 1.30	10.16	0.01	2	T1 > T0,T2
SJS (mm)	2.25 ± 0.86	4.63 ± 1.53	2.74 ± 1.38	18.74	0	2	T1 > T0,T2
Angle (°)	11.08 ± 3.32	16.24 ± 4.71	12.90 ± 3.57	9.27	0.01	2	T1 > T0
Volume (mm^3^)	683.5 ± 143.93	893.19 ± 181.11	722.64 ± 167.76	9.59	0.01	2	T1 > T0,T2

^(1)^ Statistical significance was analyzed by Friedman’s test (*p* < 0.05). ^(2)^ Adjustment for multiple comparisons: Bonferroni. AJS, anterior joint space; PJS, posterior joint space; SJS, superior joint space; T0, pre-surgical; T1, post-surgical one week; T2, 4–5 months after screw removal; SD, standard deviation.

## Data Availability

The data that support the findings of this study are available from the corresponding author upon reasonable request.
